# Nurses’ Knowledge and Anxiety Levels toward COVID-19 in Saudi Arabia

**DOI:** 10.3390/nursrep11020034

**Published:** 2021-05-10

**Authors:** Fatmah Alsharif

**Affiliations:** Medical Surgical Department, Faculty of Nursing, King Abdulaziz University, Jeddah 21589, Saudi Arabia; falsharif@kau.edu.sa

**Keywords:** coronavirus, COVID-19, epidemic, psychological impact, mental health, psychiatry, stress, anxiety, Saudi Arabia, nurse

## Abstract

*Background:* In the battle against the Coronavirus Disease 2019 (COVID-19) pandemic, medical care staff, especially nurses, are at a higher risk of encountering psychological health issues and distress, such as stress, tension, burdensome indications, and, most importantly, fear. They are also at higher risk of becoming infected and transmitting this virus. In Saudi Arabia, it was noticed that the healthcare workforce suffered from anxiety, and that this more evident in women than men. **Objective:** This study aimed to assess the knowledge of nurses regarding COVID-19 and the level of anxiety toward the COVID-19 outbreak in the current pandemic situation. *Design:* A cross-sectional design was used and a validated self-administered online questionnaire with a set of questions related to COVID-19 was distributed to 87 participating nurses. *Results:* The results showed that more than half of the nurses (71.90%) had an adequate and good knowledge about the causes, transmission, symptoms, treatment, and death rate of COVID-19. The main sources of information for the nurses were social media (51.7%) and the World Health Organization and the Ministry of Health (36.8%). *Conclusions:* The results allowed the conclusion that, though the nurses had satisfactory knowledge about COVID-19, more than 50% of them experienced mental health issues such as anxiety. To address this, along with providing more knowledge about COVID-19, nurses should be supported in managing their anxiety.

## 1. Introduction

On 2 March 2020, Saudi Arabia recorded the first case of coronavirus (COVID-19) [1]. In the battle against the new (COVID-19), nursing staff in Saudi Arabia have been confronted everyday with tremendous work tension. They are at constant high risk of becoming infected with COVID-19. As a result, they are experiencing negative feelings such as disappointment, isolation, detachment, and loss of contact with family members, which has caused a decline in their service quality and negative attitudes toward patients [2]. This has caused psychological wellbeing issues such as stress, tension, burdensome indications, sleeping disorders, frustration, anger, and, most importantly, fear. The level of psychological issues varies and may appear in any stage. These psychological wellness issues influence the nature of the care given by medical care staff, their clinical agreement, and their dynamic abilities, which could make it hard to battle COVID-19, as well as significantly affecting their prosperity and personal satisfaction. In Saudi Arabia, a cross sectional study was conducted to explore depression and anxiety levels among healthcare providers. The study showed more than half of them had depressive disorder (55.2%) and generalized anxiety disorder (51.4%). The study indicated that nurses had a significantly higher score of anxiety in comparison with other healthcare providers [3]. Therefore, it is imperative to secure the psychological wellbeing of the healthcare workforce to gain sufficient control of the current crisis [2]. In most general clinics in China, an arrangement of work shifts was set up to permit specialists to rest and avoid high work pressure [2]. This study aimed to assess the knowledge of nurses regarding COVID-19 and their levels of anxiety toward the COVID-19 outbreak in the current pandemic situation.

COVID-19 has affected everybody physically and emotionally, which may have led to vulnerability, sleep deprivation, outrage, the fear of contamination, increased consumption of liquor or cigarettes, social separation, increase in post-pressure harmful effects, nervousness issues, burdensome confusion, somatization, and loss of wellbeing [2]. For these reasons, the wellbeing framework should invest energy and consideration into supporting susceptible groups: (a) contaminated patients, their families, and the healthcare staff who care for them, (b) individuals with previous physical and mental issues, and (c) health and backing experts [2]. Arafa et al. [4] conducted a cross-sectional study among different hospitals in Saudi Arabia in April 2020. The study aimed to assess the level of psychological distress among the participants, measured as anxiety, depression, and stress. A Google survey was completed by the participants, in which areas such as their occupational features, sleeping hours, and the psychological impacts (anxiety, depression, and stress) of the COVID-19 outbreak were assessed using the Depression Anxiety Stress Scale-21 (DASS-21). The study included 426 healthcare professionals (48.4% physicians, 24.2% nurses, and 27.4% other HCWs). The results concluded that among the total sample of 426 participants, 69% had depression, 58.9% had anxiety, 55.9% had stress, and 37.3% had inadequate sleep. Another study by Huang et al. [5] conducted an elucidating cross-sectional investigation in Japan from 7 to 14 February 2020. The goals of the investigation were to assess the mental condition of the healthcare staff responding against COVID-19 and to provide a hypothetical premise for psychological support. The study included 246 participants. The self-evaluation scale for tension (SAS) and the self-appraisal scale for post-traumatic stress (PTSD-SS) were applied. In total, 230 surveys were recovered (a recovery rate of 93.5%). The investigation included 43 men (18.7%) and 187 women (81.3%) aged 20 to 59 years (32.6 ± 6.2); of these, 70 were specialists (30.4%) and 160 were attendants (69.6%) [5]. The finding determined that the rate of unease among the healthcare workforce was 23.04% (53/230), and the nervousness score was 42.91 ± 10.89. Among the respondents, the rates of extreme nervousness, moderate tension, and mild unease were 2.17% (5/230), 4.78% (11/230), and 16.09% (37/230). The unease rate was higher among females than among men, and female nervousness scores were also higher than men’s (43.78 ± 11.12) versus (39.14 ± 9.01), (t = −2.548, *p* = 0.012). The tension rate among medical attendants was higher than that among doctors, and the unease scores of the attendants were higher than those of the specialists. The pressure issue rate among the healthcare faculty was 27.39% (63/230) and the pressure issue score was 42.92 ± 17.88. The pressure score of female healthcare workers was higher than that of men [5].

Healthcare staff have a high occurrence of uneasiness and stress. Foundational consideration ought to be given to the mental abilities of the healthcare workforce, including attendants. Additionally, mental mediation groups should be created to give counsel on unease and stress to workers in the healthcare sector [6].

## 2. Materials and Methods

### 2.1. Study Design

A quantitative descriptive cross-sectional approach was used in this study of nurses in Saudi Arabia.

### 2.2. Sample and Setting

Inclusion criteria were (a) nurses from the medical and surgical departments of King Abdul Aziz University Hospital Jeddah and (b) nurses working in the hospital departments dealing with the admission and hospitalization of COVID-19 patients, (c) who were able to read and speak English. Data were collected using convenience sampling of nursing employees. Due to COVID-19, the research was conducted via online survey, avoiding direct contact with the participants. The online platform for the study data collection was available from 1 July 2020 to 15 July 2020. The data were collected from a university hospital located in Jeddah, Saudi Arabia. The online data collection facilitated access to the target participants during the COVID-19 pandemic and complied with the strict hospital policy to reduce exposure. The sample size was calculated using G*power 3.1 software, which is recommended when conducting descriptive studies. At least 85 participants were needed for this analysis. Additionally, sample size was calculated by Raosoft software which recommend minimum sample size was 70, based on a 50% response rate, a 90% confidence interval (CI), and a 10% margin of error.

### 2.3. Measures

A validated questionnaire that was previously used in Iran was used [7]. This questionnaire tool was designed based on a literature review by WHO regarding developing respiratory diseases including COVID-19. Demographic and clinical characteristics were collected. The questionnaire comprised three different sections. The first part contained basic demographic information (age, gender, educational level, work experience) of the participants.

The second section included 22 questions regarding anxiety and knowledge of COVID-19. This segment of the questionnaire, according to the recommendations of the WHO about the virus, included items such as the knowledge of participants toward COVID-19 sources, symptoms, mode of transmission, mortality rate, and treatment (four of these questions were “Yes/No” or “I do not know” responses, and eight questions were multiple choice).

The third section contained the Lickerts scale of anxiety levels among participants concerning infection of their families with COVID-19. The knowledge scores ranged from 7 to 24. A score of less than the cutoff (<16) was labeled as adequate knowledge, and equal to or above the cutoff (≥16) was deemed to be good knowledge. The validation and reliability test of the data collection instrument revealed an internal consistency of 70%.

### 2.4. Ethical Considerations

Ethical approval was obtained from the King Abdulaziz University Hospital in Jeddah, Kingdom of Saudi Arabia. Participation in this study was voluntary and the identities of the participants were not recorded anywhere on the questionnaire. The researcher explained the aim of the study through the online survey. Participants answered the questions after providing online consent, and they could withdraw at any time while answering the questions. Ethics approval was granted by the hospital’s ethics board (Reference No 361-20).

### 2.5. Data Analysis

The results were computed and analyzed using Statistical Package for the Social Sciences (SPSS) 26.0 version (IBM Inc., Chicago, IL, USA). To describe the variables, descriptive statistics were used, including mode, median, and standard deviation; these were computed using SPSS 26.0. The *t*-test and ANOVA at the 0.05 significance level were used to compare different factors between different groups. Cronbach’s alpha was used for the overall knowledge and anxiety score. A Cronbach’s alpha coefficient greater than 0.70 was considered acceptable.

## 3. Results

### 3.1. Participants

A total of 87 participants (staff nurses) were included in this study. Most participants (53; 60.9%) were aged between 31 and 40 years old, and 26.4% (23 nurses) were aged between 41 and 50 years old. About 82 (94.3%) were female. A total of 48 (55.2%) respondents had a nursing diploma. Additionally, 47.1% of respondents had a work history of between 5 and 15 years, as indicated in Table 1.

### 3.2. Knowledge Level of Participants

The majority of the respondents (58.6% of the sample study) knew that the 2019 novel coronavirus caused the disease. Of the respondents, 60.9% had information about the coronavirus before its Saudi Arabia outbreak. A total of 36.8% of respondents received their information about the coronavirus from the WHO and the Ministry of Social Affairs. About 30% of the respondents rated their information about coronavirus as an 8 out of 10. Of the respondents, 94.3% said that COVID-19 is infectious, 2.3% said that headaches are a symptom of COVID-19, 48.3% of the sample study said that COVID-19 is like MERS, 46% of them reported that COVID-19 is like SARS, 33.3% agreed that bats are the source of Coronaviruses, 97.7% of them stated that hand washing is the Coronavirus prevention method, and 10.3% reported that the rate of COVID-19-induced mortality is 2%. A majority (87.4%) of the sample study stated that supportive treatment is the routine treatment for COVID-19, 95.4% said that 2–14 days is the incubation period of this virus, 72.4% study stated that the coronavirus is transmitted through the air via sneezes and coughs, 59.8% said that incidence can be reduced by greater involvement of treatment staff, and 85.1% of them said that there is a need for more people to be trained by the medical staff.

A majority (87.4%) of the respondents did not know anyone in their family that had been infected with COVID-19. Of the respondents, 96.6% of them had not been infected and 44.8% of the sample study had received a COVID-19 diagnostic test (Table 2).

### 3.3. Level of Anxiety of Participants

**Figure 1:**
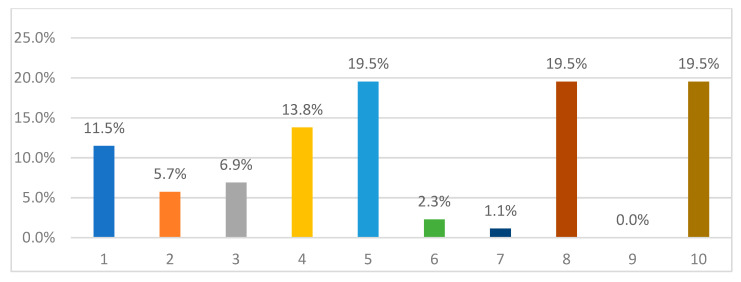
Anxiety levels.

A fifth (20%) of the study sample rated their anxiety level about infection as 10 out of 10 (Figure 1). A significant minority (35.6%) rated their worry about their loved ones being infected as 10 out of 10. The mean was 5.7 on the 10th Likert scale. That mean fell in the sixth group, which means that the level of anxiety was equal to six.

### 3.4. Level of Knowledge and Anxiety of Participants

To present the level of knowledge and anxiety, the means of both were computed. The mean overall score was 12.94 out of 18, which means that the percentage of knowledge reached 72%. These results are displayed in Table 3.

For Anxiety: The mean was 5.7 on the 10th Likert scale. That mean fell in the sixth group, which means that the level of anxiety was equal to six, as indicated in Table 4.

Table 5 indicates that the *p*-value of the test was less than 0.05; thus, we reject the null hypothesis and accept the alternative one, denoting that there was a significant difference in knowledge about COVID-19 between educational levels with a level of confidence of 95%.

## 4. Discussion

COVID-19 is a life-threatening infectious agent that has spread across the world and has become a global concern. This disease was first identified in Wuhan on 12 December 2019 [8]. Nurses, in particular, come into direct contact with infected patients and play a critical role in infection prevention. Assessing the level of nurses’ knowledge about COVID-19 could be an important step in controlling the disease in Saudi Arabia, which has one of the highest infection rates among Arab countries. This study aimed to assess the knowledge of nurses regarding COVID-19 and their levels of anxiety toward the COVID-19 outbreak in the current pandemic situation. The current research study of 87 nurses revealed their concerns about COVID-19 infection in themselves and their families. In a study conducted during the COVID-19 outbreak in China, Haung and Zhao discovered that healthcare staff had poor sleep quality and higher levels of anxiety than the general population [9]. The results of the study are consistent with previous study conducted in Yemen which reported that healthcare providers including nurses had an adequate knowledge and moderate level of anxiety [10].

Another study by Wang et al. [11] investigated the impact of the COVID-19 outbreak. They conducted an elucidating cross-sectional examination in the initial 14 days of the COVID-19 outbreak and applied an emotional wellbeing assessment from 31 January to 2 February 2020. The aspects of the investigation were as follows: to assess the pervasiveness of mental manifestations and to distinguish hazard and defensive factors corresponding to mental pressure [11]. The study included 1210 participants from 194 cities. An online survey was conducted using a snowball sampling technique to gather data from the participants. Most of the respondents were females (67.3%) between 21.4 and 30.8 years old (53.1%), married (76.4%), living with three to five individuals (80.7%), and with children (67.4%) [11]. The fear of being infected, the uncertainty around controlling the disease, and the scarcity of medical facilities across the country are all possible causes of high anxiety.

This study found that our sample of nurses had good knowledge of COVID-19 infection during the current outbreak, with more than half of them having good knowledge (more than the cut-off point). Having sufficient knowledge can indicate the effective dissemination of COVID-19 information across various media. These findings support a report that found that healthcare workers have a clear understanding of MERS and a positive attitude toward it [12]. Our findings revealed that nurses obtain their information from a variety of sources, including reliable websites, the World Health Organization, and the Ministry of Health. Other research found that participants learned about infectious diseases mostly from the internet and television, which is consistent with our results [13]. There was a significant difference in knowledge about COVID-19 between different educational levels. Another Saudi Arabian study on students from various majors and educational backgrounds found no significant impact of age or education level on their knowledge, which is not consistent with our findings [14].

The results of this study can be utilized to plan interventions to improve the emotional wellness of susceptible populations during the COVID-19 pandemic. Ruiz-Grosso et al. [15] directed an enormous public investigation on mental distress in the Spanish population during COVID-19, in which they applied an online self-report survey from 31 January to 10 February 2020. The poll fused standards for assessing pressure-related issues and explicit fears, as indicated by the ICD-11, socio-segment information, and the major impacts of COVID-19 Peritraumatic Distress Index (CPDI) according to the recurrence of tension, despondency, explicit fears, psychological changes, shirking and impulse conduct, and loss of social usefulness in the most recent week. A total 35% of the sample experienced mental trouble, of which 29.29% were classed as having mild to direct distress and 5.14% as having extreme pain. Females manifested more mental distress than men according to CPDI scores of (SD) = 24.87 (15.03) versus 21.41 (15.97), *p* < 0.001. Individuals under 18 years old presented less mental trouble according to CPDI scores (SD) = 14.83 (13.41). Two defensive variables may clarify the low degree of mental pain in those under 18 years old: a moderately low incidence of pessimism in this age group and restricted exposure to the pandemic because of home isolation [15]. Individuals aged 18 to 30 years and those older than 60 years showed high levels of mental pain, with CPDI scores of (SD) = 27.76 (15.69) and CPDI scores (SD) = 27.49 (24, 22), respectively. The populace aged between 18 and 30 years old presented data on informal organizations, triggering pressure. Given that the most noteworthy mortality rates have been reported in older populations, it is reasonable to assume that people in this age group are struggling with the most extreme mental impacts. Additionally, populations with a more elevated level of schooling will in general have more prominent mental pain, likely because of more attention paid to risks to their wellbeing. Traveling workers reported a more elevated level of misery compared to people in different occupations, with CPDI scores (SD) of 31.89 (23.51), F = 1602.501, *p* < 0.001 [15].

## 5. Limitations

The participant number is somewhat low, which represents the major limitation in the study. This was due to the timeframe that been approved by the hospital IRB as well as the shortage of nursing staff. The fact that only nurses participated in this study limits the generalizability of its results. There may have been information bias due to the use of an online self-reported measure. Thus, replies mainly depended upon trustworthiness and may have been affected by participants’ willingness to participate. The potential sample might also limit the generalizability of study.

## 6. Study Implications

It is very beneficial to consider mixed-method designs in the future studies attempting to describe psychological impacts on nurses and medical staff. One of the greatest advantages of applying mixed-method designs is that more in-depth information can be gathered regarding feeling, emotion, and cultural psychological impacts.

## 7. Conclusions

The COVID-19 pandemic in Saudi Arabia could place our wellbeing framework at great risk, forcing public authorities to enforce isolation and other extreme measures. This situation would have adverse effects on the healthcare workforce and the susceptible populace and might set off pressure, dread, disarray, outrage, dissatisfaction, stress, weariness, dejection, shame, tension, misery, blame, sorrow, and self-destruction. Therefore, the arrangement of psychological wellness groups for emergency mediation, utilization of computerized stages, online correspondence, telemedicine interviews, and utilization of brief instruments for the identification of emotional wellbeing issues would be valuable and helpful for the difficulties currently faced within Saudi Arabia.

## Figures and Tables

**Table 1:** Sociodemographic characteristics of the study’s participants.

Factor		*n*	%
Gender	Male	5	5.7
	Female	82	94.3
Age	20–25	1	1.1
	26–30	3	3.4
	31–40	53	60.9
	41–50	23	26.4
	51 and above	7	8.0
Education level	Nursing diploma	48	55.2
	Bachelor’s degree in nursing	38	43.7
	Master’s degree‘ or above	1	1.1
Work experience	1–5 years	6	6.9
	5–15 years	41	47.1
	15–25 years	31	35.6
	More than 25 years	9	10.3

Total *n* = 87. This table uses descriptive statistics: frequency (*n*) and percentage (%).

**Table 2:** Percentage of COVID-19 distribution.

	Yes, *n* (%)	No, *n* (%)
Been infected with COVID-19	3 (3.4%)	84 (96.6%)
Received a COVID-19 diagnostic test	39 (44.8%)	48 (55.2%)

Total *n* = 87. This table uses descriptive statistics: Frequency (*n*) and percentage (%).

**Table 3:** Level of knowledge and anxiety of participants (*n* = 87).

Descriptive Statistics					
	*n*	Min	Max	Mean	Overall Score
What Is the Rate of Your Anxiety Level about Infection? (1–10)	87	1	10	5.7011	6%
Knowledge	87	1	18	12.9425	71.90%

**Table 4:** Level of anxiety of participants (*n* = 87).

Rate Weight	From	To
1	1	1.9
2	1.9	2.8
3	2.8	3.7
4	3.7	4.6
4	4.6	5.5
6	5.5	6.4
7	6.4	7.3
8	7.3	8.2
9	8.2	9.1
10	9.1	10

**Table 5:** Educational level of participants (*n* = 87).

	*n*	Mean	Std. Deviation	Std. Error	F	Sig.
Nursing diploma	48	11.7917	2.19243	0.31645	16.523	<0.01
Bachelor’s degree	38	14.5000	2.37953	0.38601		
Master’s degree or above	1	9.0000				
Total	87	12.9425	2.65609	0.28476		

## Data Availability

The data is available from the authors on request.
